# Mechanisms of Cellular Internalization of Quantum Dot® Conjugated Bone Formation Mimetic Peptide CK2.3

**DOI:** 10.3390/nano8070513

**Published:** 2018-07-09

**Authors:** Vrathasha Vrathasha, Karl Booksh, Randall L. Duncan, Anja Nohe

**Affiliations:** 1 Department of Biological Sciences, University of Delaware, Newark, DE 19716, USA; vrathash@udel.edu (V.V.); rlduncan@udel.edu (R.L.D.); 2 Department of Chemistry and Biochemistry, University of Delaware, Newark, DE 19716, USA; kbooksh@udel.edu

**Keywords:** Bone, Osteoporosis, CK2.3, Qdot®s, Fluorescence imaging, FTIR

## Abstract

Osteoporosis is a debilitating skeletal disorder that is characterized by loss of bone density over time. It affects one in two women and one in four men, age 50 and older. New treatments that specifically drive bone formation are desperately needed. We developed a peptide, CK2.3, that acts downstream of the bone morphogenetic protein receptor type Ia and it induces osteogenesis in-vitro and in-vivo. However, its mechanism of action, especially its mode of uptake by cells remains unknown. To demonstrate CK2.3 internalization within a cell, we conjugated CK2.3 to Quantum Dot®s (Qdot®s), semiconductor nanoparticles. We purified CK2.3-Qdot®s by size exclusion chromatography and verified the conjugation and stability using UV/VIS and Fourier transform infrared spectroscopy. Our results show that CK2.3 was conjugated to the Qdot®s and the conjugate was stable for at least 4 days at 37 °C. Moreover, CK2.3-Qdot®s exerted biological response similar to CK2.3. Addition of CK2.3-Qdot®s to cells followed by confocal imaging revealed that CK2.3-Qdot®s were internalized at 6 h post stimulation. Furthermore, using pharmacological inhibitors against endocytic pathways, we demonstrated that CK2.3-Qdot®s were internalized by caveolae. These results show for the first time that the novel peptide CK2.3 is taken up by the cell through caveolae mediated endocytosis.

## 1. Introduction

Osteoporosis is a silent disease due to its gradual progression of loss in bone mass and deterioration of the microarchitecture of the bone tissue. It predisposes patients to an increased risk of fractures at either the hip, spine, or wrist [1,2]. Approximately 10 million Americans are diagnosed with osteoporosis and another 44 million are at an increased risk of a fracture due to their low bone density. Women are more likely to be diagnosed with osteoporosis than the combined incidence of breast cancer, heart attack, and stroke [3].

Majority of the existing treatments for osteoporosis such as recombinant parathyroid hormone (PTH) [4], bisphosphonates [5], and selective estrogen receptor modulators (SERMS) [6,7] have multiple side effects [8] and new therapeutics are desperately needed.

Our lab developed a peptide, CK2.3, by using the protein kinase 2 (CK2) phosphorylation consensus sequence found on bone morphogenetic protein receptor type Ia (BMPRIa), the phosphorylation motif is conserved and distinct from other consensus sequences of protein kinases that have been characterized so far [9]. CK2 is a serine/threonine kinase, it is a highly conserved, ubiquitously expressed tetrameric structure with two catalytic subunits (α and/or α’) and two regulatory subunits (β). CK2α/α’ is active both in its free state as well as in the tetrameric form, CK2β appears to only regulate the substrate specificity of the tetrameric complex [10,11]. CK2 has a host of physiological targets and some phospho-proteomic databases predict that 20% of all phospho-proteome are substrates of CK2 [9]. Furthermore, CK2 has been implicated to play a role in adipogenic, myogenic, hematopoietic and osteogenic differentiation [12,13]. CK2.3 acts downstream of BMPRIa and functions by inhibiting CK2 from interacting with BMPRIa [14,15].CK2.3 stimulation activates the BMP signaling pathway and induces the formation of mineral nodules in C2C12 cells (murine myoblasts) [16,17], primary calvarial osteoblasts, and bone marrow stromal cells (BMSCs) [18]. Moreover, systemic injection of CK2.3 into 8-week-old female C57BL/6J mice increased trabecular bone mineral density, number of trabeculae, trabecular thickness, and decreased trabecular spacing [18]. However, very little is known regarding the mechanism of CK2.3 mediated induction of osteogenesis, especially the time frame and mechanism of its internalization.

In order to determine these parameters, a fluorescent probe was designed by using Quantum Dot®s (Qdot®s) [19]. Qdot®s are semiconductor nanoparticles [20,21] about 10–20 nm in diameter and they fluoresce under UV light. Their high photostability, brightness, and narrow range of emission makes them ideal for labelling and real-time tracking applications [19,22,23,24].

The Qdot®s used in this study were coated with carboxyl groups, which were used to conjugate it to the amino groups of the peptide CK2.3. There are five lysine amino acids in the CK2.3 sequence, and it also has the N-terminal amino group. *N*,*N*′-Dicyclohexylcarbodiimide (DCC) was used for the coupling [25].

Conjugated CK2.3-Qdot®s were separated from non-conjugated reactants by size exclusion chromatography (SEC). The separation of the sample was determined by UV/VIS spectroscopy and the conjugation was confirmed by Fourier transform infrared (FTIR) spectroscopy. FTIR was also used to determine the stability of CK2.3-Qdot®s. CK2.3-Qdot®s were stable for at least 4 days at 37 °C. To characterize the activity of CK2.3-Qdot®s in-vitro, C2C12 cells were used as the model cell line, as it is one of the most commonly used mesenchymal stem cell- derived cell line to study osteoporosis in-vitro [26,27,28]. Furthermore, C2C12 cells is an ideal cell line to study the role of BMPRIa in inducing osteogenesis, since it is reported that BMPRIa is expressed in C2C12 cells [29] and in fact, BMPRIa is the key mediator in inducing C2C12 differentiation into osteoblasts [30]. Using von Kossa assay, we determined that CK2.3 retained its mineralization capabilities in the conjugated CK2.3-Qdot®s. Cell labelling of C2C12 cells treated with CK2.3-Qdot®s, followed by imaging using confocal laser scanning microscope showed that CK2.3 was taken up at 6 h post stimulation. Additionally, treatment of C2C12 cells with pharmacological inhibitors against clathrin coated pits (CCPs) and caveolae showed that caveolae mediated endocytic pathway is essential for CK2.3 internalization into C2C12 cells. 

Significance of the study: Currently prescribed drugs against osteoporosis (e.g., PTH, bisphosphonates, SERMS) have failed to find a lasting treatment for this disease, and in many cases, have led to adverse side effects [8]. We have previously reported that CK2.3 is a novel and potential bone-formation inducing drug. However, its mechanism of action has not been studied. In this paper, we developed a novel bio-imaging probe. Using this probe, coupled with confocal microscopy we were able to determine the time frame of CK2.3 uptake. Additionally, we determined the detailed mechanism of CK2.3 internalization, which is mediated by caveolae. The results were surprising, since Qdot®s in general are taken up either by pinocytosis [31] or CCPs [32,33,34,35]. This further demonstrates that CK2.3 is directing the uptake and localization within the cell.

## 2. Materials and Methods

### 2.1. Materials

#### 2.1.1. CK2.3 Peptide

CK2.3 (1.915 μM) was designed using the CK2 phosphorylation sequence found on BMPRIa, which was identified using the prosite search and it was ordered from GenScript (Piscataway, NJ, USA). 16 amino-acid long (2.246 kDa) Antennapedia homeodomain signal sequence [36] is incorporated at the N terminal of CK2.3, to aid in the cellular uptake of the peptide. Altogether, CK2.3 consists of 29 amino acids and has a molecular weight of 3.6 kDa.

#### 2.1.2. Carboxyl Quantum dot®s (Qdot®s)

Qdot®s (Qdot® 525 ITK^™^ carboxyl quantum dots, catalog # Q21341MP, 8 μM) were purchased from Life technologies, (Carlsbad, CA, USA), the Qdot®s used here are made up of cadmium selenide core with zinc sulfide shell, coated with non-toxic carboxyl groups and are excitable at 488 nm. The Qdot®s have an ellipsoid core/shell with diameters of 6 nm (minor axis) by 12 nm (major axis). The hydrodynamic diameters were measured by high-performance size exclusion chromatography with a major axis to be 18 nm for carboxylic acid coated Qdot®s [37].

### 2.2. Methods

#### 2.2.1. Conjugation of CK2.3 to Qdot®s

To conjugate CK2.3 to Qdot®s, a solution mixture consisting of 2 μL of Qdot®s (8 μM), 10 μL of CK2.3 (1.915 μM), and 2 μL of 10 × PBS were mixed in 84 μL of dimethyl sulfoxide (DMSO) purchased from Fisher Scientific (Pittsburg, PA, USA). 2 μL of *N,N′*-Dicyclohexylcarbodiimide (DCC) (36 mg dissolved in 1 mL of DMSO) from Sigma-Aldrich (St. Louis, MO, USA) was added to initiate the conjugation. As control, eight different reactions consisting of all possible combinations of reactants were used. The reactions were carried out at room temperature for 30 min. After 30 min, 200 μL of 1 × PBS pH 7.4 was added to quench the reaction and the samples were immediately put on ice. We added equal moles of Qdot®s and CK2.3, to ensure a 1:1 conjugation ratio.

#### 2.2.2. Size Exclusion Chromatography (SEC)

Conjugated CK2.3-Qdot®s were separated from unspecific reactants based on SEC. Sephadex G-50 fine beads were purchased from Sigma-Aldrich (St. Louis, MO, USA). 2.5 g of Sephadex G-50 beads were suspended in 50 mL of distilled water and allowed to swell overnight, the slurry was then packed into 3 mL syringe columns and centrifuged at 2000× *g* rpm for 5 min to tightly pack the beads and remove excess water from the columns. 300 μL of the conjugation solution, as well as the eight other control reactions were gradually added to their respective filtration columns. Later, 100 μL of distilled water was gently added one at a time to each filtration columns, to collect 30 fractions of samples from each column. The samples were then analyzed using UV/VIS spectroscopy to identify the fraction containing the conjugated CK2.3-Qdot®s. On an average, we obtained about 160 nM of CK2.3-Qdot®s solution per conjugation reaction, as determined using the standard curve. Absorbance of CK2.3-Qdot®s is background corrected by subtracting the absorbance value of the control PBS sample.

#### 2.2.3. UV/VIS Spectroscopy

UV/VIS spectra were collected by drop-casting 2 μL (×3 times) of sample onto the pedestal of NanoDrop® (ND-1000 Spectrophotometer). UV/VIS spectra were gathered by plotting absorbance of sample against its respective range of wavelength (220–300 nm). The concentration of the conjugation was calculated using the standard curve. For this curve, the absorbance of Qdot®s at 223 nm was plotted against known concentrations of Qdot®s. After conjugation, the optical density of the conjugate was determined at 223 nm and eventually the concentration of CK2.3-Qdot®s was calculated using the standard curve.

#### 2.2.4. FTIR Spectroscopy

Mid-infrared spectra were collected in specular reflectance mode by drop-casting 5 μL (×3 times) of sample onto gold-coated round coverslips from Substrata (Kitchener, ON, Canada) and the sample was analyzed using Bruker Optics vertex FTIR spectrometer (Bruker Optics Inc., Billerica, MA, USA) equipped with Hyperion 2000 microscope and liquid nitrogen cooled Mercury-Cadmium-Telluride (MCT) detector. Each spectrum consists of 100 scans averaged at a 4 cm^−1^ resolution. OPUS v6.0 was used for spectral acquisition. Essential FTIR software was used to view and baseline correct each spectrum.

#### 2.2.5. Cell Culture

C2C12 cells (murine myoblast cells) were purchased from American Type Culture Collection (Manassas, VA, USA). Cells were grown in Dulbecco’s Modified Eagle’s Medium (DMEM) (Hy-Clone, Pittsburgh, PA, USA) supplemented with 20% (*v*/*v*) heat in-activated fetal bovine serum (FBS) (Gemini Bio-products, West Sacramento, CA, USA), 1% (*v*/*v*) penicillin/streptomycin (Hy-clone, Pittsburgh, PA, USA). Phenol-free DMEM media (Hy-Clone, Pittsburgh, PA, USA) supplemented only with 1% (*v*/*v*) penicillin/streptomycin (Hy-clone, Pittsburgh, PA, USA), but no FBS. 1 × Trypsin (Cellgro, Manassas, VA, USA) was used to detach cells from flasks during sub-culturing.

#### 2.2.6. Von Kossa Assay

C2C12 cells were grown in 24-well plate at a cell density of 1 × 10^4^ cells/well for two days in DMEM media containing 20% FBS and 1% penicillin/streptomycin and at the end of the 2nd day, cells were serum starved for 18 h with DMEM media containing 1% penicillin/streptomycin and without FBS. Cells were then either left unstimulated or stimulated with 100 nM of CK2.3 and CK2.3-Qdot®s on day 1 and then again on day 3, cells were supplemented with DMEM media containing 10% FBS and 1% penicillin/streptomycin following 24 h of treatment. After 5 days, cells were fixed using 4.4% (*w*/*v*) paraformaldehyde from Sigma-Aldrich (St. Louis, MO, USA) for 20 min and washed with cold 1 × PBS pH 7.4. 5% (*w*/*v*) silver nitrate from Chem-Impex international (Wood Dale, IL, USA) dissolved in diH_2_O was applied to each well and the plate was placed under the UV light for 1 h. Cells were then washed with diH_2_O and were allowed to dry overnight. Twelve random images of the cells were taken per well using Zeiss Axiovert 10 microscope at 5×/12 Achrostigmat objective and images were analyzed using ImageJ software (NIH, Bethesda, MD, US.). Images were first converted to 8 bit and threshold was set to the positive control. The same threshold was used for all treatments. Mineralized areas were quantified using the analyzing particles plugin function of ImageJ. Data was analyzed using one-way Anova and Tukey-Kramer post hoc statistical test.

#### 2.2.7. Immunofluorescence Labelling of C2C12 Cells and Analysis

C2C12 cells were grown in 35 mm dishes containing 18 mm × 18 mm square coverslips at a cell density of 1 × 10^4^ cells/well for two days in DMEM media containing 20% FBS and 1% penicillin/streptomycin and at the end of the 2nd day, cells were serum starved with phenol-free DMEM media supplemented with 1% penicillin/streptomycin, but without FBS for 18 h.

(a) Visualization of CK2.3-Qdot®s uptake within cells. C2C12 cells were either left unstimulated or stimulated with 100 nM of CK2.3-Qdot®s for 2 h, 6 h, 12 h, and 18 h. After each time point, plasma membrane was stained using 100 μL (10 ng/μL) of FM™ 4-64FX membrane dye (catalog # F34653) from Fisher Scientific (Pittsburg, PA, USA) diluted in 1× PBS pH 7.4 and incubated for 1 min on ice, followed by fixation of the cells using 4.4% (*w*/*v*) paraformaldehyde purchased from Sigma-Aldrich (St. Louis, MO, USA) for 20 min and permeabilized with 0.05% (*w*/*v*) saponin from Sigma-Aldrich (St. Louis, MO, USA) diluted in diH_2_O and incubated for 10 min on ice, and later the nucleus was stained using 100 μL (0.5 ng/mL) of Hoechst (catalog # 23491-45-4) from Sigma-Aldrich (St. Louis, MO, USA). The coverslips were then mounted on the slides using an anti-fading mixture called as Airvol. It is made up of 20 g polyvinyl alcohol from Chemistry Connection LLC (Conway, AR, USA) diluted in 80 mL of diH_2_O, the mixture is stirred at 60–70 °C for 2 h. Followed by the addition of 39.6 mL of Glycerol, 2 g of n-propyl gallate from Sigma-Aldrich (St. Louis, MO, USA), and 0.4 mL of 0.2 M Tris (pH 8.5) from Fisher Scientific (Pittsburg, PA, USA). The mixture is centrifuged at 18,000× *g* for 30 min and stored in −20 °C for long term storage. Slides were imaged using Zeiss LSM 710 at 20×/0.8 and 63×/1.4 oil Plan-Apochromat objectives.

(b) Confirmation of conjugation between CK2.3 and Qdot®s. C2C12 cells were either left unstimulated or stimulated with 100 nM of CK2.3-Qdot®s for 12 h. After 12 h, cells were fixed using 4.4% (*w*/*v*) paraformaldehyde purchased from Sigma-Aldrich (St. Louis, MO, USA) for 20 min and permeabilized with 0.05% (*w*/*v*) saponin from Sigma-Aldrich (St. Louis, MO, USA) diluted in diH_2_O and incubated for 10 min on ice. Cells were blocked with 3% (*w*/*v*) protease-free Bovine Serum Albumin (BSA) from Fisher Scientific (Pittsburg, PA, USA) diluted in 1× PBS pH 7.4, at room temperature for 1 h. Following blocking, cells were labelled for Antennapedia homeodomain using 100 μL of mouse monoclonal anti-Antp 4C3 from Developmental Studies Hybridoma Bank (University of Iowa) diluted in the ratio of 1:100 in 3% protease-free BSA, at room temperature for 1 h. The primary antibody was then stained against using 100 μL (1 μg/μL) of fluorescently tagged-secondary antibody, donkey anti-mouse IgG H&L (Alexa Fluor® 568, Catalog # ab175472) from Abcam (Cambridge, MA, USA) diluted in the ratio of 1:500 in 3% protease-free BSA, at room temperature for 1 h. Later the nucleus was stained using 100 μL (0.5 ng/mL) of Hoechst (catalog # 23491-45-4) from Sigma-Aldrich (St. Louis, MO, USA). The coverslips were then mounted on the slides using Airvol [38]. Specificity of donkey anti-mouse Alexa Fluor® 568 was determined by labeling unstimulated cells with only the fluorescent secondary antibody. Slides were imaged using Zeiss LSM 710 at 63×/1.4 Plan-Apochromat oil objective.

(c) Co-localization of CK2.3-Qdot®s with either endogenous Adaptin α or Caveolin-1 proteins. C2C12 cells were either left unstimulated or stimulated with 100 nM of CK2.3-Qdot®s for 6 h. After 6 h, cells were fixed using 4.4% (*w*/*v*) paraformaldehyde purchased from Sigma-Aldrich (St. Louis, MO, USA) for 20 min and permeabilized with 0.05% (*w*/*v*) saponin from Sigma-Aldrich (St. Louis, MO, USA) diluted in diH_2_O, for 10 min on ice. Cells were blocked with 3% (*w*/*v*) protease-free Bovine Serum Albumin (BSA) from Fisher Scientific (Pittsburg, PA, USA) diluted in 1× PBS pH 7.4, at room temperature for 1 h. Following blocking, cells were either labelled for Caveolin-1 or Adaptin α proteins using 100 μL (2.5 ng/μL) of purified mouse anti-Caveolin-1 (catalog # 610494) and purified mouse anti-Adaptin α (catalog # 610501); respectively from BD Transduction Laboratories™ (San Jose, CA, USA), diluted in the ratio of 1:100 in 3% protease-free BSA, at room temperature for 1 h. The primary antibody was stained against using 100 μL (20 ng/μL) of fluorescently tagged-secondary antibody, goat anti-mouse IgG (H + L) (Alexa Fluor® 633, Catalog # A21052) from Fisher Scientific (Pittsburg, PA, USA) diluted in the ratio of 1:500 in 3% protease-free BSA, at room temperature for 1 h. Later the nucleus was stained using 100 μL (0.5 ng/mL) of Hoechst (catalog # 23491-45-4) from Sigma-Aldrich (St. Louis, MO, USA). The coverslips were then mounted on the slides using Airvol [38]. Specificity of donkey anti-mouse Alexa Fluor® 568 was determined by labeling unstimulated cells with only the fluorescent secondary antibody. Slides were imaged using Zeiss LSM 710 at 63×/1.4 Plan-Apochromat oil objective.

#### 2.2.8. Inhibition of CCPs- and Caveolae-Mediated Endocytosis

C2C12 cells were grown in 35 mm dishes containing 18 mm × 18 mm square coverslips at a cell density of 1 × 10^4^ cells/well for two days in DMEM media containing 20% FBS and 1% penicillin/streptomycin and at the end of the 2nd day, cells were serum starved with phenol-free DMEM media supplemented with 1% penicillin/streptomycin, but without FBS for 18 h. Next, we used chlorpromazine from Cayman chemical (Ann Arbor, MI, USA) to inhibit CCPs-mediated endocytosis. Chlorpromazine is a cationic amphiphilic drug and it functions by inhibiting the formation of CCPs by reversible translocation of clathrin and its adapter proteins from plasma membrane to intracellular vesicles [39,40]. C2C12 cells were treated with 5 μm of Chlorpromazine in DMEM media supplemented with 10% FBS and 1% penicillin/streptomycin for 24 h [41]. Lovastatin from TCI America (Portland, OR, USA) was employed to prevent caveolae-mediated endocytosis, it functions by depleting cholesterol by metabolic inhibition of cholesterol synthesis [41]. C2C12 cells were treated with 50 μm of Lovastatin in phenol-free DMEM without FBS and supplemented only with 1% penicillin/streptomycin for 24 h [41,42]. Methyl-β-cyclodextrin from TCI America (Portland, OR, USA) was used to inhibit both CCPs- and caveolae-mediated endocytosis, MβCD is a cyclic oligomer of glucopyranoside and it works by reversibly removing steroid out of the plasma membrane thereby preventing cholesterol-mediated endocytosis [40]. C2C12 cells were treated with 100 μm of methyl-β-cyclodextrin in phenol-free DMEM without FBS and supplemented only with 1% penicillin/streptomycin for 24 h [43]. After 24 h of treatment of cells with inhibitors against endocytosis, C2C12 cells were stimulated with 100 nM of CK2.3-Qdot®s for 18 h. After 18 h, cells were fixed using 4.4% (*w*/*v*) paraformaldehyde purchased from Sigma-Aldrich (St. Louis, MO, USA) for 20 min and permeabilized with 0.05% (*w*/*v*) saponin from Sigma-Aldrich (St. Louis, MO, USA) diluted in diH_2_O, for 10 min on ice. Later the nucleus was stained using 100 μL (0.5 ng/mL) of Hoechst (catalog # 23491-45-4) from Sigma-Aldrich (St. Louis, MO, USA). The coverslips were then mounted on the slides using Airvol [38]. Slides were imaged using Zeiss LSM 710 at 63×/1.4 Plan-Apochromat oil objective.

## 3. Results

### 3.1. Development of a Biologically Active and Fluorescently Tagged CK2.3-Qdot®s Probe

**Figure 1:**
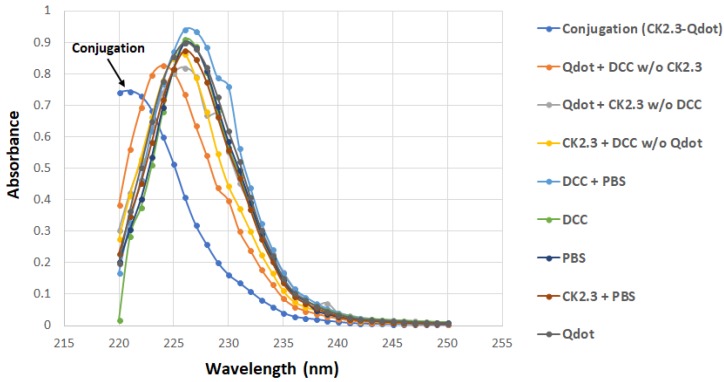
UV/VIS graph representing the absorbance of the CK2.3-Qdot®s fraction, along with the eight other control samples between wavelength 220–250 nm. Conjugated CK2.3-Qdot®s has a distinctive peak compared to its controls, and the absorbance band between 220–225 nm is indicative of secondary amides [45].

The carboxyl group on the Qdot®s was conjugated to the amino group of CK2.3 in the presence of DCC [25] as described in the method section. Conjugated CK2.3-Qdot®s were isolated by performing SEC; Sephadex G-50 fine beads were used as the gel filtration resin [44]. The samples eluted off the columns were analyzed using UV/VIS spectroscopy. UV/VIS spectra of CK2.3-Qdot®s fraction and the spectra of the eight other control fractions were obtained. The spectra of CK2.3-Qdot®s depicted a distinctive peak compared to its respective controls. Furthermore, a band was observed between 220–225 nm in case of CK2.3-Qdot®s fraction [45] (Figure 1). For each sample, we collected 30 fractions. Unconjugated Qdot®s and non-specific reactants are eluted off the filtration column and can be found in different fractions. 

**Figure 2:**
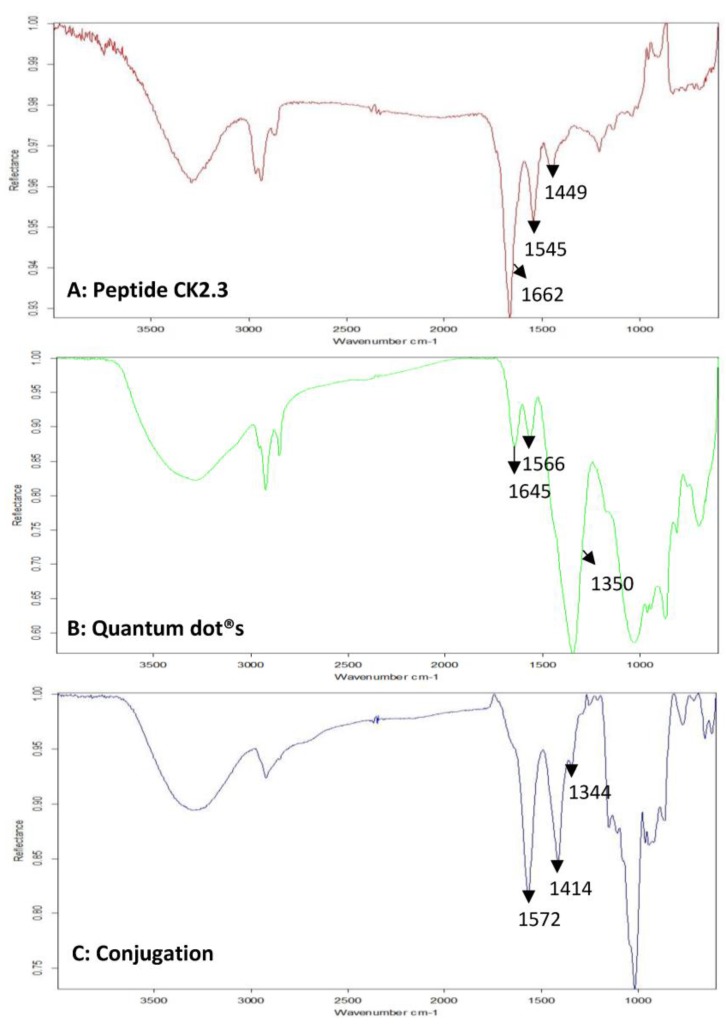
Mid-infrared spectra of samples, drop-casted on gold-coated coverslips were collected using Bruker Optics vertex FTIR spectrometer. FTIR spectra of (**A**): Peptide CK2.3, (**B**): Qdot®s, and (**C**): CK2.3-Qdot®s.

**Figure 3:**
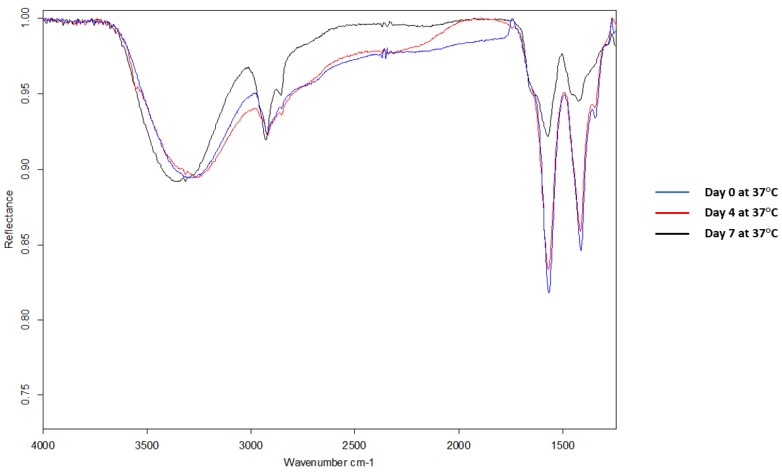
Mid-infrared spectra of samples, drop-casted on gold-coated coverslips were collected using Bruker Optics vertex FTIR spectrometer. FTIR spectra of CK2.3-Qdot®s depicting its stability at 37 °C over the course of day 0, day 4, and day 7. FTIR spectra’s of CK2.3-Qdot®s at day 4 and day 7 are about 94.44% and 44.44% similar to day 0 spectra; respectively.

Next, FTIR was used to verify the conjugation between CK2.3 and Qdot®s. In the spectra of CK2.3 (Figure 2A), peaks were observed at 1664 cm^−1^ and 1546 cm^−1^ which represent amide I bond and amide II bond respectively [46] and ~1430 cm^−1^ represented the asymmetric bending of proteins [47]. In the case of Qdot®s (Figure 2B), peaks were observed at 1556 cm^−1^ and 1355 cm^−1^, which signify the carboxylate (carboxylic acid salt) peaks [48,49]. The peak at 1645 cm^−1^ represented the OH bending of absorbed water [49]. With respect to the conjugation (Figure 2C), new peaks appeared at 1572 cm^−1^, 1414 cm^−1^, and 1344 cm^−1^. Absorption peaks of amide I and amide II moved to a lower frequency [50,51]. Conjugation moves the amide groups to lower frequency [52] due to resonance effect [53]. Thus, confirming the conjugation between CK2.3 and Qdot®s. Next, we determined the stability of the conjugated CK2.3-Qdot®s at 37 °C (Figure 3) over the course of seven days using FTIR. By comparing the FTIR spectra’s of CK2.3-Qdot®s at day 0 (blue), day 4 (red), and day 7 (black); we noticed that the spectra at day 0 and day 4 were about 94.44% similar, however, at day 7, the amide I and amide II bonds appeared to have a prominent shoulder at 1658 cm^−1^ and 1466 cm^−1^; respectively. Presence of a shoulder suggests that some of the CK2.3 are no longer conjugated to Qdot®s and thus, CK2.3-Qdot®s were breaking into their separate components. However, even at day 7 about 44.44% of CK2.3 and Qdot®s are still conjugated.

**Figure 4:**
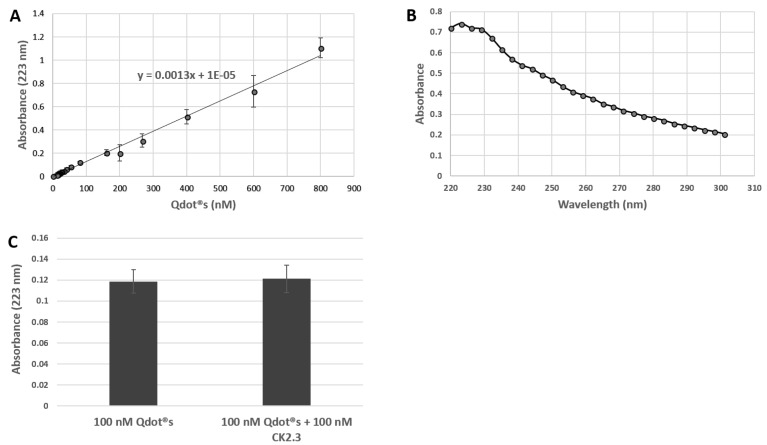
UV/VIS spectra were collected by drop-casting 2 μL of sample onto the pedestal of ND-1000 Spectrophotometer. (**A**): Standard curve of Qdot®s representing the absorbance of known concentrations of Qdot®s (0–800 nM) at 223 nm; (**B**): Sample UV/VIS spectra of unconjugated Qdot®s with maximum wavelength at 223 nm; (**C**): Graphical representation of absorbance at 223 nm by 100 nM of Qdot®s, compared to solution mixture containing 100 nM of Qdot®s and 100 nM of CK2.3.

In order to expose C2C12 cells to equimolar amounts of either CK2.3 or CK2.3-Qdot®s, we determined the concentration of the CK2.3-Qdot®s fraction. We determined the absorbance of unconjugated Qdot®s at 223 nm wavelength to its respective known concentrations (0–800 nm) (Figure 4A) and subsequently using the standard curve to correlate it to the absorbance of CK2.3-Qdot®s [54]. Using this approach, we determined the concentration of CK2.3-Qdot®s and used this data to calibrate our assays when we compared the activity of CK2.3-Qdot®s to CK2.3. Additionally, in (Figure 4B) we show the sample UV/VIS spectra of unconjugated Qdot®s, which appears to have a maximum wavelength at 223 nm. Furthermore, in (Figure 4C) we demonstrate that addition of 100 nM of peptide CK2.3 does not change the absorbance of 100 nM of Qdot®s at 223 nm, thus the absorbance of Qdot®s overpowers the absorbance of CK2.3.

**Figure 5:**
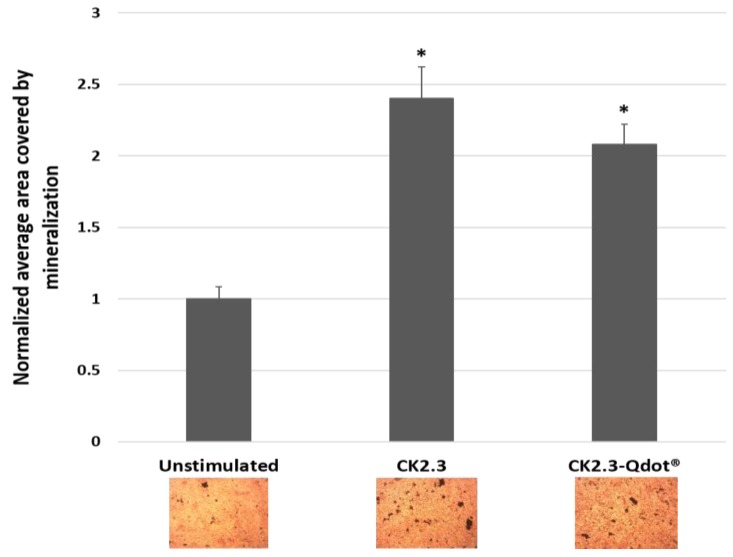
Mineralization levels in unstimulated C2C12 cells and cells treated with 100 nM of CK2.3 and CK2.3-Qdot®s was determined using von Kossa assay. Data was analyzed using one-way anova and Tukey-Kramer post hoc statistical test. * denotes statistical significance at p-value of 0.05 compared to unstimulated cells. CK2.3 and CK2.3-Qdot®s induced 2.4 ± 0.221 and 2.07 ± 143 fold more mineralization compared to unstimulated cells; respectively.

CK2.3 induces significant mineralization at 100 nM in C2C12 cells [16,17], therefore, we confirmed that the conjugated CK2.3-Qdot®s retained their biological activity using von Kossa assay. For this assay, C2C12 cells were either left untreated or treated with 100 nM of CK2.3 and CK2.3-Qdot®s and stained for mineralization using 5% silver nitrate. Von Kossa assay is performed to visualize calcium deposits. The silver ions of the silver nitrate solution, bind to the anions such as phosphates, sulfates, or carbonates of calcium salts and in presence of a strong reducing light such as UV, the silver salts are reduced to form dark brown or black metallic silver staining, which corresponds to calcium deposits [55]. Cells treated with 100 nM of CK2.3 and CK2.3-Qdot®s induced significant mineralization, about 2.4 ± 0.221 and 2.07 ± 143 fold more calcium deposits compared to unstimulated cells; respectively (Figure 5). This result confirmed that the mineralization inducing activity of CK2.3 is retained in the conjugated CK2.3-Qdot®s.

### 3.2. Time-Dependent Uptake of CK2.3-Qdot®s by C2C12 Cells

**Figure 6:**
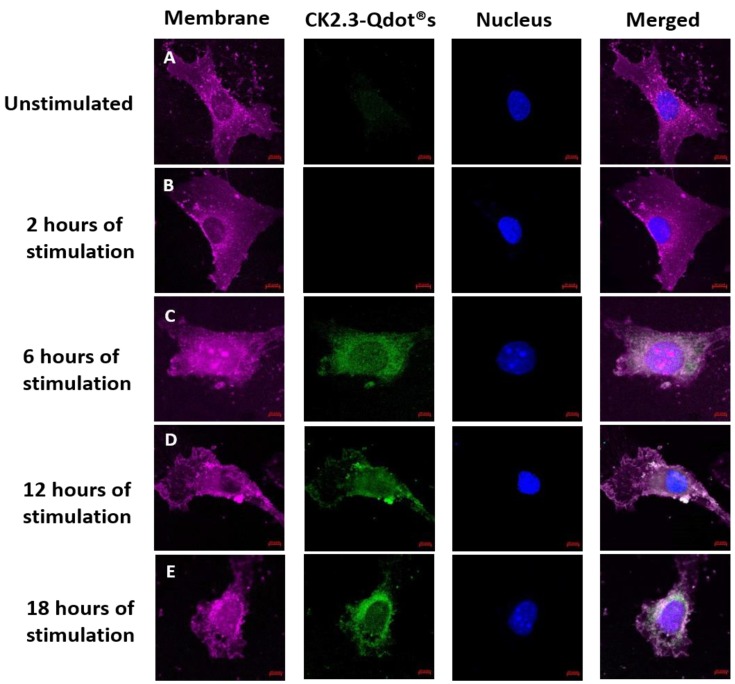
Stimulation of C2C12 cells with CK2.3-Qdot®s (green) for 2 h, 6 h, 12 h, and 18 h. Following stimulation, cells were fixed using 4.4% PFA and the plasma membrane (magenta) and nucleus (blue) of the cells were stained using FM™ 4-64FX and Hoechst; respectively. Images taken at 20×/0.8 objective depict the interaction of CK2.3-Qdot®s with the plasma membrane in (**A**): unstimulated cells, and following (**B**): 2 h, (**C**): 6 h, (**D**): 12 h, and (**E**): 18 h of stimulation.

**Figure 7:**
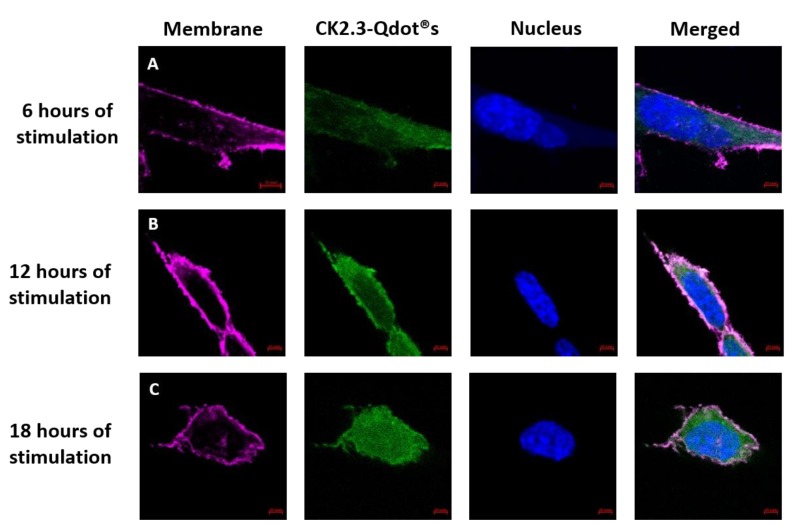
Uptake of CK2.3-Qdot®s (green) by C2C12 cells at 6 h, 12 h, and 18 h. Following stimulation, cells were fixed using 4.4% PFA and the plasma membrane (magenta) and nucleus (blue) of the cells were stained using FM™ 4-64FX and Hoechst; respectively. Images of cells taken using 63×/1.4 objective at (**A**): 6 h, (**B**): 12 h, and (**C**): 18 h show the localization of CK2.3-Qdot®s within the cell.

CK2.3 induces osteogenesis and bone formation in-vitro [16,17] and in-vivo [16,18], however its mechanism of action in inducing osteogenesis still needs to be understood. To define the function of CK2.3 in-vitro, CK2.3 was conjugated to Qdot®s, which fluoresces when excited at 488 nm. Qdot®s are 18 nm in size and the surface chemistry of the Qdot®s is compatible with the aqueous environment of the cell [19]. This conjugation allowed us to track the movement of CK2.3 via Qdot®s within a cell, using confocal laser scanning microscopy. C2C12 cells were either left unstimulated or stimulated with 100 nM of CK2.3-Qdot®s for 2 h, 6 h, 12 h, and 18 h. After each time point, the plasma membrane of the cells were stained, fixed and imaged using confocal microscopy. Qdot®s can be excited at 488 and their fluorescence can be measured using confocal microscopy. Presence of Qdot®s (green) on or within the plasma membrane (magenta) of C2C12 cells were first detected at 6 h post stimulation (Figure 6C, images were taken using objective 20×/0.8 and Figure 7A, images were taken using objective 63×/1.4). At 12 h, increased accumulation of CK2.3-Qdot®s was detected inside the cell (Figure 7B) and at 18 h, CK2.3-Qdot®s was distributed throughout the cytoplasm of the cell (Figure 7C). Figure 7 depicts images of cross sections taken through the cell, demonstrating that CK2.3-Qdot®s are localized intracellular.

**Figure 8:**
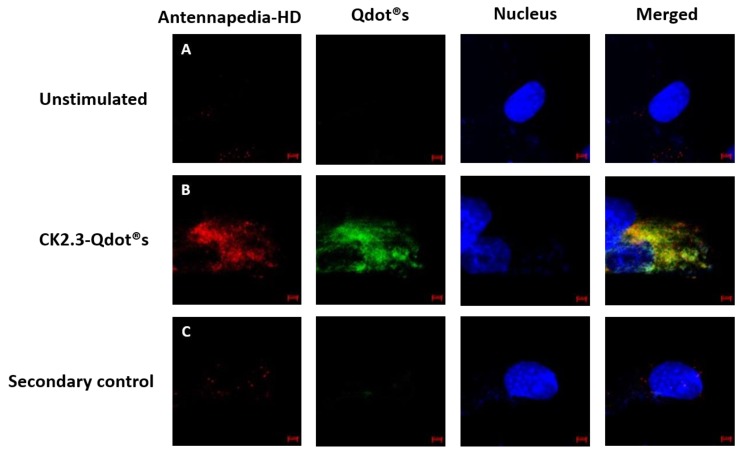
Co-localization of Antennapedia homeodomain signal sequence (red), incorporated within the sequence of CK2.3, and Qdot®s (green). (**A**): C2C12 cells were either left untreated or (**B**): treated with 100 nM of CK2.3-Qdot®s for 12 h. Later the cells were fixed and the nucleus (blue) was stained using Hoechst. Images of the cells treated with CK2.3-Qdot®s show that signals from CK2.3 and Qdot®s co-localize almost 100%. (**C**): As a negative control, unstimulated cells were stained using only the fluorescent secondary antibody to determine their specificity and lack of staining in the cell shows that the antibody is specific against the antigen.

To further verify the conjugation between CK2.3 and Qdot®s, we stained for the Antennapedia homeodomain. The Antennapedia homeodomain signal sequence was incorporated in the peptide sequence of CK2.3. The third α-helix of the Antennapedia homeodomain, a 16-amino acid long peptide, is well known for its ability to translocate across the plasma membrane of a cell in a receptor-independent manner [56,57,58,59]. Cells were either left unstimulated or stimulated with 100 nM of CK2.3-Qdot®s. After fixation, cells were subsequently stained for Antennapedia homeodomain signal sequence and images were collected using confocal microscopy. Results obtained showed that the signals from Qdot®s (green) and CK2.3 (red), co-localized (yellow) (Figure 8B). This data demonstrates, that the size exclusion chromatography completely separates the derivatized Qdot®s from the non-derivatized Qdot®s. It also confirms that CK2.3 is bound to the Qdot®s in the cell. Additionally, as a negative control, unstimulated cells were stained using only the fluorescent secondary antibody to determine their specificity and lack of staining in the cell shows that the antibody is specific against the antigen (Figure 8C).

### 3.3. Caveolae Mediated Endocytosis Is Utilized by CK2.3-Qdot®s for Its Internalization

**Figure 9:**
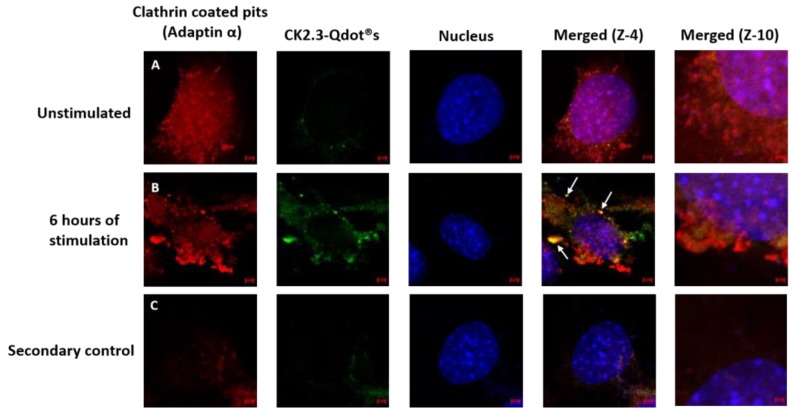
Mechanism of endocytic uptake of CK2.3-Qdot®s. (**A**): C2C12 cells were either left untreated or (**B**): treated with 100 nM of CK2.3-Qdot®s for 6 h. Later the cells were fixed and the nucleus (blue) was stained using Hoechst. Cells were stained against Adaptin α (red), which slightly co-localized with CK2.3-Qdot®s (green) as seen in zoom-4 image. (**C**): As a negative control, unstimulated cells were stained using only the fluorescent secondary antibody to determine their specificity and lack of staining in the cell shows that the antibody is specific against the antigen.

**Figure 10:**
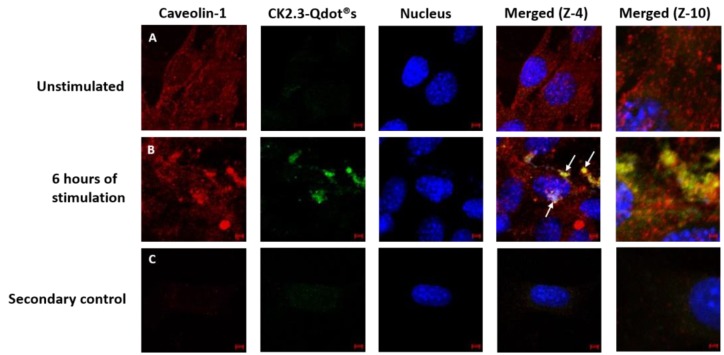
Mechanism of internalization of CK2.3-Qdot®s. (**A**): C2C12 cells were either left untreated or (**B**): treated with 100 nM of CK2.3-Qdot®s for 6 h. Later the cells were fixed and the nucleus (blue) was stained using Hoechst. In case of caveolae, Caveolain-1 (red) and CK2.3-Qdot®s (green) co-localize to a higher extent as clearly seen in zoom-4 and zoom-10 images. (**C**): As a negative control, unstimulated cells were stained using only the fluorescent secondary antibody to determine their specificity and lack of staining in the cell shows that the antibody is specific against the antigen.

Thus far, we have confirmed that CK2.3-Qdot®s were taken up by C2C12 cells as early as 6 h post stimulation. Next, we investigated the mechanism of its internalization, either via CCPs- or caveolae-mediated endocytosis. C2C12 cells were either left unstimulated or stimulated with 100 nM of CK2.3-Qdot®s for 6 h. Following stimulation, one set of cells were stained for the Adaptin α of CCPs- and another set of cells were stained for Caveolin-1, a marker for caveolae-mediated endocytosis. In case of CCPs (Figure 9), there was some co-localization (yellow) between Adaptin α (red) and CK2.3-Qdot®s (green) (Figure 9B, zoom-4). However, in case of caveolae mediated endocytosis (Figure 10), we observed higher intensities of co-localization (yellow) between Caveolin-1 (red) and CK2.3-Qdot®s (green) (Figure 10B, zoom-4 and zoom-10). These images show that CK2.3-Qdot®s localized with these domains, however they do not show which pathway is essential for CK2.3-Qdot®s internalization into C2C12 cells. 

**Figure 11:**
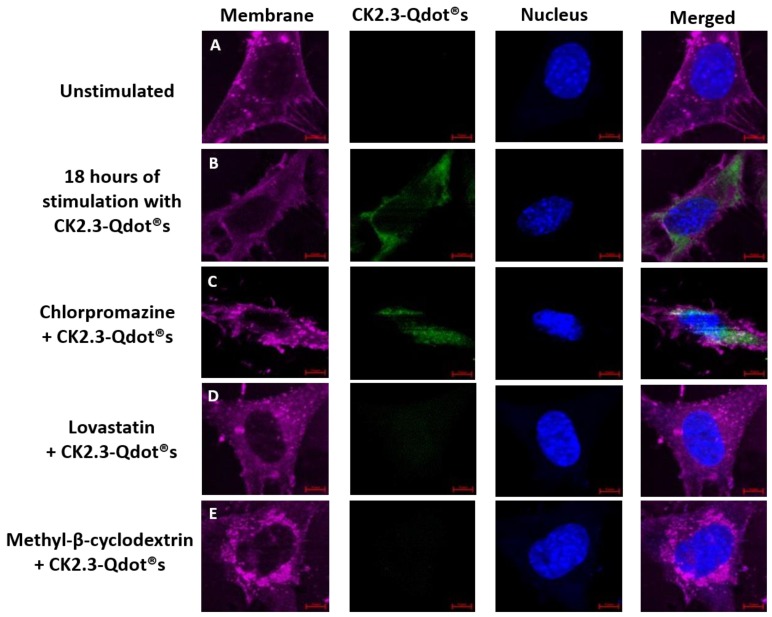
Pharmacological inhibitors were used against endocytic pathways. Chlorpromazine, lovastatin, and methyl-β-cyclodextrin inhibitors were employed to inhibit CCPs-, caveolae-, and both endocytic pathways; respectively. (**A**): C2C12 cells were either left untreated or (**B**): treated with 100 nM of CK2.3-Qdot®s for 18 h. Following stimulation, CK2.3-Qdot®s appeared to be internalized within the cell. (**C**): Treatment of cells with chlorpromazine did not affect the endocytosis of CK2.3-Qdot®s, whereas, (**D**): treatment of cells with lovastatin and (**E**): methyl-β-cyclodextrin completely inhibited the internalization of CK2.3-Qdot®s.

Next, we employed pharmacological inhibitors against specific endocytic pathways. We used chlorpromazine and lovastatin to inhibit CCP- and caveolae mediated endocytosis; respectively [41]. To inhibit both endocytic pathways we used methyl-β-cyclodextrin (MβCD) [60]. We selected these inhibitors since it was shown that these inhibit caveolae and clathrin mediated endocytosis in C2C12 cells and have been used previously [41,43]. C2C12 cells were either left unstimulated or stimulated with the pharmacological inhibitors for 24 h. The following day, cells were further treated with 100 nM of CK2.3-Qdot®s for 18 h, as maximum intensity of Qdot®s were observed at 18 h post stimulation within the cell. Cells were fixed, and images were collected using a confocal microscope (Figure 11). Cells treated with CK2.3-Qdot®s showed internalization of CK2.3 within the cell (Figure 11B). Similarly, chlorpromazine did not affect the uptake of CK2.3-Qdot®s and showed its internalization within the cell (Figure 11C). However, treatment of cells with lovastatin and MβCD prevented CK2.3-Qdot®s from entering C2C12 cells (Figure 11D,E). Based on this result, CK2.3-Qdot®s are internalized within C2C12 cells via caveolae mediated endocytosis and in fact Antennapedia homeodomain is the one driving its translocation across the plasma membrane into the cytoplasm of the cell. Furthermore, BMPRIa is reported to aggregate in the caveolar microdomains [61,62], also Caveolin-1 is crucial in the activation of BMPRIa downstream signaling [63,64,65].

## 4. Discussion

In this paper we report for the first time the development of a biologically active fluorescent CK2.3 analog. We chose Qdot®s as our fluorescent label due to their photostability and brightness compared to traditional organic dyes, fluorescent proteins, and lanthanide chelates [19,22,23]. Moreover, Qdot®s are commonly used in confocal imaging of protein interactions and dynamics. Drawbacks for their use include the size of the nanoparticle (18 nm) compared to smaller fluorescent organic dyes, and therefore they can change the activity and biological properties of the protein they are conjugated to. However, in our studies, the peptide CK2.3 retained its biological activity and directed its uptake within the cells. The use of DCC for crosslinking followed by size exclusion chromatography is an established method [25,44]. We previously used this method to conjugate calcitriol to Qdot®s [66]. With respect to the conjugation reaction, the carboxyl group on the Qdot®s is conjugated to the amino group in CK2.3. Furthermore, we employed size exclusive chromatography to separate the conjugated CK2.3-Qdot®s from other undesired conjugates. This was performed manually and therefore was very time consuming and resulted in low yield of the conjugate. We only obtained 100 μL of ~150 nM solution per conjugation. Lack of a concentrated CK2.3-Qdot®s solution, limited the use of the method. In order to perform more experiments, one will have to upscale the experiment. The conjugation between CK2.3 and Qdot®s was determined by FTIR Spectroscopy. We performed a time series experiment, by incubating CK2.3-Qdot®s for 1 week at 37 °C followed by FTIR analysis. These results showed that the peptide stayed conjugated to Qdot®s for at least 4 days. FTIR requires to dry the sample on gold surface and the drying itself may alter the sample. However, drying samples prior to analysis using FTIR is a commonly practiced technique [67]. Next, we determined the biological activity of CK2.3-Qdot®s in cells by performing von Kossa assay. To ensure that the peptide is conjugated to Qdot®s and not released over a period of time, we determined the co-localization of the peptide with the Qdot®s. We stimulated cells with CK2.3-Qdot®s and used an antibody that recognizes the Antennapedia homeodomain, which is present in the peptide. As our data showed, after 12 h of incubation with CK2.3-Qdot®s, almost 100% of the peptide co-localizes with the Qdot®s. These experiments confirm that CK2.3 is conjugated to the Qdot®s and it is biologically active within the cells. 

Next, we investigated the mechanism of uptake of CK2.3-Qdot®s into the cells. Clathrin dependent endocytosis is the most well characterized method of endocytosis, it is receptor-dependent and can internalize molecules of up to 200 nm in size [59]. The process of clathrin mediated internalization occurs in five stages: initiation, cargo selection, coat assembly, scission and uncoating [68]. In the first step, clathrin relies on adaptor proteins and complexes such as AP2 to be recruited to the plasma membrane. Adaptor proteins consists of two large adaptin proteins such as Adaptin α; which was used here to stain for clathrin-dependent endocytosis. Whereas, caveolae mediated endocytosis involves invagination of plasma membrane that are rich in cholesterol and sphingolipids and stain strongly for Caveolin-1 [69]. It can internalize molecules that are 50–80 nm in size from the lipid raft region of the plasma membrane through a receptor-independent mechanism [59]. Both membrane regions, CCPs and caveolae are indicated in the BMP2 signaling pathway. However, recent experiments point to the importance of caveolae for the activation of BMP receptor signaling [63,64]. Indeed, it is shown that BMP2 specifically binds to BMP receptors found in caveolae and not in CCPs [61]. Therefore, CK2.3 taken up through caveolae mediated endocytosis specifically mimics the BMP2 signaling pathway activation. Previously, we have reported that CK2.3 induces Smad-1,5 and 8 signaling, as well as the activation of ERK pathway [15,18]. However, the signaling pathways activated by CK2.3-Qdot®s in inducing mineralization will need to be determined and compare it to the CK2.3 mediated signaling pathways.

It has been reported that the Antennapedia homeodomain signal sequence incorporated in CK2.3 sequence, mediates cellular uptake in a receptor-independent mechanism [59,70]. However, conjugating CK2.3 to Qdot®s may lead to its uptake via a different pathway. For example, stimulation of J774.A1 cells with COOH-Qdot®s resulted in detection of fluorescence within the cell as early as 30 min post treatment [71]. It is reported that, anionic nanoparticles are mainly targeted to the clathrin mode of endocytic pathway for cellular uptake [32,33,34,35]. Our study with the pharmacological inhibitors against endocytic pathways, along with the co-localization experiments demonstrate that CK2.3-Qdot®s were taken up by caveolae and that the Antennapedia homeodomain was driving the translocation of CK2.3 across the plasma membrane and into the cell.

Our long-term goal is to visualize the distribution and localization of CK2.3-Qdot®s within living organisms using non-invasive, real-time in-vivo fluorescence imaging. Some of the limitations associated with using conventional organic dyes are short lifetimes, photodegradation, limited fluorescence brightness, along with intrinsic tissue autofluorescence [22,23,24]. Hence, we are unable to use the Antennapedia homeodomain antibody to track CK2.3. However, these shortcomings can be overcome by using the superior photophysical properties of Qdot®s. Several groups have used Qdot®s for in-vivo and deep tissue imaging [72,73], thus far human tumors have been implanted in mice to track cancer cells using Qdot®s during metastasis [74,75,76]. Therefore, Qdot®s are promising and have the potential to be used as a bio-imaging fluorophore to visualize and track the movement of CK2.3 within bone tissue of osteoporotic animal models. However, there are some cytotoxicity concerns involved with the usage of Qdot®s which is due to the chemical composition of its cadmium and selenide metal core [22,23]. But, cytotoxicity studies conducted using Qdot®s reveal it to be non-toxic for short term experiments [37,71]. 

In conclusion, we have developed a biologically active, fluorescently tagged CK2.3 imaging probe. We have shown that CK2.3 is conjugated to the Qdot®s, and the bioconjugate is stable for at least 4 days at 37 °C and remains conjugated within the cell for at least a day. We have also confirmed that the biological activity of CK2.3 is retained in the CK2.3-Qdot®s conjugate. Furthermore, using confocal microscopy, we have determined that CK2.3 is taken up by C2C12 cells as early 6 h post stimulation and it is internalized via caveolae mediated endocytosis. It is significant that CK2.3 co-localizes with Caveolin-1 since it is reported that BMPRIa receptors aggregate in the caveolar microdomains [61,62] and it plays a crucial role in the activation of BMPRIa downstream signaling [65]. Therefore, internalization of CK2.3 through the caveolae shows that CK2.3 is localized exactly where BMPRIa is reported to be predominantly available, and hence it is functioning as per its intended design.
